# Recent Advances in Artificial Intelligence and Tactical Autonomy: Current Status, Challenges, and Perspectives

**DOI:** 10.3390/s22249916

**Published:** 2022-12-16

**Authors:** Desta Haileselassie Hagos, Danda B. Rawat

**Affiliations:** DoD Center of Excellence in Artificial Intelligence and Machine Learning (CoE-AIML), Howard University, Washington, DC 20059, USA

**Keywords:** tactical autonomy, autonomous systems, artificial intelligence, military, defense applications, aerospace, machine ethics, cybersecurity, trustworthiness, explainability

## Abstract

This paper presents the findings of detailed and comprehensive technical literature aimed at identifying the current and future research challenges of tactical autonomy. It discusses in great detail the current state-of-the-art powerful artificial intelligence (AI), machine learning (ML), and robot technologies, and their potential for developing safe and robust autonomous systems in the context of future military and defense applications. Additionally, we discuss some of the technical and operational critical challenges that arise when attempting to practically build fully autonomous systems for advanced military and defense applications. Our paper provides the state-of-the-art advanced AI methods available for tactical autonomy. To the best of our knowledge, this is the first work that addresses the important current trends, strategies, critical challenges, tactical complexities, and future research directions of tactical autonomy. We believe this work will greatly interest researchers and scientists from academia and the industry working in the field of robotics and the autonomous systems community. We hope this work encourages researchers across multiple disciplines of AI to explore the broader tactical autonomy domain. We also hope that our work serves as an essential step toward designing advanced AI and ML models with practical implications for real-world military and defense settings.

## 1. Introduction

Emerging technologies, such as robotics and autonomous systems, are providing opportunities for potentially revolutionizing society [1]. Advanced autonomous systems are paving the way for scientific breakthroughs and disruptive technological innovations across several domains of science [2]. Autonomous systems are a network of intelligent systems capable of independently performing complex tasks, making intelligent decisions without explicit human intervention, and other operations management and control systems [3,4]. The most recent developments in modern autonomous systems are becoming increasingly crucial for a wide variety of potential military and defense applications, including air surveillance systems, privacy, cybersecurity, missile defense, the aerospace industry, etc.

**Context and motivation**. Research scientists from the civilian, defense, and military communities are working through the complexities to determine the best ways of implementing advanced AI and autonomous systems for industry and real-world applications. Leveraging AI, ML, and other related advanced technology domains for autonomous systems is a tactically game-changing strategy for modern autonomous systems.

Modern and cutting-edge AI and ML techniques have been increasingly used in the military and defense domain for a variety of successful applications, including cybersecurity [5], maritime security [6,7], critical infrastructure protection [8,9], and other domains of significant societal and technological importance. The potential of advanced AI systems can be used in ways that positively impact military and defense technologies. AI can be used in the military setting to evaluate the collected data and provide operational planning and strategic support, accelerating decision-making processes. In addition to this, AI systems can be designed and deployed to be used in strategic, political, operational, and tactical levels of warfare.

In the context of political and strategic levels, AI systems can be used to dynamically destabilize hidden enemies and defend against various forms of adversarial attacks in real time. At the tactical level, however, AI can provide faster and improved situational awareness for unmanned systems to reduce their vulnerability to attacks. It can also efficiently automate threat detection by identifying suspicious patterns and potentially dangerous activities. However, despite the autonomy advances across a broad range of areas over the past few decades, several technical and practical challenges continue to significantly limit the deployment and wide adoption of modern autonomous systems. Some of the critical challenges that need to be tackled are addressed in Section 4, Section 5 and Section 6. Therefore, it is essential to develop modern tactical autonomous systems with minimum supervision or involvement from humans that substantially improve the state-of-the-art and reduce cognitive workloads and increase functions, improve, and maintain multi-domain situational awareness, enhance overall maneuverability and mobility, effectively enable force protection, support proactive cyber defense, etc.

Motivated by the increasing interest and popularity of autonomy, this paper presents a comprehensive and technical survey of the fundamental concepts and principles of tactical autonomy, with a focus on cutting-edge AI and ML approaches that have not been adequately addressed in previous research works. To the best of our knowledge, this is the first work that addresses the important current trends, strategies, fundamental challenges, tactical complexities, and future research directions on tactical autonomy.

**Contribution**. The major contributions of our paper are summarized as follows.

- We introduce the fundamental concepts of tactical autonomy and its potential across a broad range of applications.
- We capture an understanding of the notion of tactical autonomy in the context of military and defense settings.
- To the best of our knowledge, we are the first to provide the important current trends, strategies, fundamental challenges, tactical complexities, and future research directions on tactical autonomy.
- We present a work that can serve as an important step towards designing advanced and innovative AI and ML models with practical implications for real-world military and defense applications.
- We present the fundamental and long-standing challenges of tactical autonomy.

**Outline**. The rest of this paper is organized as follows. Section 2 provides a brief history, major milestones, ethical aspects, and levels of tactical autonomy. The different AI techniques that can be used to advance tactical autonomy capabilities are presented in Section 3. The need for trusted AI and mission autonomy is described in Section 4. The broad collaborations between platforms and the associated technical challenges are briefly described in Section 5. Section 6 presents the state-of-the-art methods for human–machine teaming and the challenges associated with the current approaches. Section 7 briefly describes cybersecurity for tactical autonomy and its fundamental challenges. An overview of the risks and inherent challenges of tactical autonomous systems is discussed in detail in Section 8. Finally, in Section 9, we conclude the paper and discuss potential future works. The abbreviations section lists the abbreviations used in this paper.

## 2. Background

The literature on autonomous systems has been broadly studied in many research works. The notion of autonomy has different contexts, and it has evolved significantly over the past few years. For example, the concept of autonomy in [10] is about a delegated task. The various aspects and dimensions of the delegation are explained in detail in [10]. Generally, autonomy in the context of intelligent systems focuses on developing intelligent decision-making systems that can physically operate autonomously in complex tactical environments with some degree of self-governance [11]. In this section, we only provide background on the works related explicitly to the history, ethical aspects, properties of autonomy, regulation, and levels of tactical autonomy.

### 2.1. Brief History and Major Milestones of Tactical Autonomy

**Figure 1:**
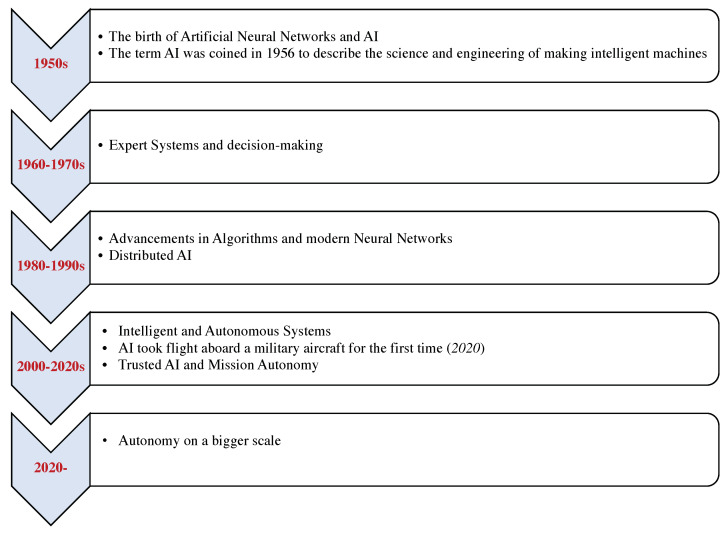
Brief history and milestones of tactical autonomy.

According to the Air Force Research Laboratory (AFRL), tactical autonomy is a term associated with modern autonomous systems acting with delegated and bounded authority of humans in support of tactical, short-term actions related to a longer-term strategic vision. In recent years, considerable interdisciplinary research has arisen on tactical autonomy for a broad range of applications. The military has long been interested in advancing the capabilities of robotics and autonomous operations. The Department of Air Force (DAF) and the Department of Defense (DoD) are pushing to conduct innovative autonomy research focused on tactical autonomy that will help transition research into practical applications. In addition, the United States AFRL is strongly prioritizing ongoing research efforts of digitally transforming tactical autonomy, particularly in the military domain, to better enable the warfighter against American adversaries. The brief history and significant milestones of tactical autonomy are depicted in Figure 1.

**Tactical Decision-Making**. Decision-making systems employ advanced models that make predictions about complex environments. Since many of these models are data-driven, autonomous systems should be able to acquire more data about the complex environments in which they operate and accordingly adapt their underlying behavior in real-time. The demand for robust and effective tactical decision-making of intelligent autonomous systems in noisy, dynamic, and realistic environments is rising rapidly. However, one of the most critical challenges is designing tactical decision-making models and supporting frameworks for autonomous systems. For example, the complexity and dynamic interaction with other road users, the complex diversity of environments, and the uncertainty in the sensor information make tactical decision-making for autonomous driving extremely difficult [12].

A general framework that combines planning and deep reinforcement learning (DRL), which can be used for tactical decision-making agents for autonomous driving systems, is described in detail in [12]. The framework’s performance is evaluated on two conceptually different scenarios of highway driving [12]. Tactical decision-making algorithms are designed to handle unforeseen environmental situations and unpredicted adversarial attacks. Processes for tactical decision-making systems can be modeled either as probabilistic (i.e., when uncertainties are included) or fully deterministic (i.e., when uncertainties are not included). Planning and decision-making in uncertainty are critical in robotics and autonomous systems. Therefore, when designing automated decision-making models and algorithms, it is important to take the various sources of uncertainty into account [13]. partially observable Markov decision process (POMDP) is a general mathematical framework employed to model decision-making tasks under uncertainty [13]. However, designing efficient approaches capable of formulating uncertainty-aware tactical decision-making tasks, such as POMDP, as well as solving its computational complexity, were not adequately addressed in previous works. Hence, as explained in Section 3, different strategies based on advanced AI/ML approaches are required to enhance the process of tactical decision-making tasks in complex and realistic environments.

### 2.2. Ethical Aspects of Autonomy

The ethical aspects of autonomy are complex challenges for AI researchers. The development and applications of modern AI-based systems are proliferating in both academia and industry. As a result, motivated by the vast improvement of speed and efficiency in the decision process, decision-making in various aspects of our daily lives is being fully delegated to AI/ML-driven algorithms. However, many important questions about the relationship between autonomy and ethics, social impact, regulations, autonomy governance, ethical implications, and capabilities of such autonomous technologies and activities have not been adequately addressed in previous studies. Therefore, exploring the safety and ethical dimensions of AI-based fully autonomous technologies enables us to acknowledge the ethical ramifications of current and future potential developments in advanced machine autonomy. Furthermore, an accurate and efficient investigation of machine intelligence’s ethics could facilitate identifying potential problems with existing ethical theories and their role in real-world environments in general. A detailed discussion of the significance of machine ethics, the study of ethical theory, and the ethical ramifications of autonomous intelligent machines are found in [14]. The research work on [14] also suggests that modern algorithms can be designed to mimic human ethical decision-making.

**Machine ethics**. As AI-driven decision-making becomes more prevalent across a wide range of fields, new and significant issues about its applicability [15], ethical dimensions, and the consideration of fundamental aspects in the design of decision-making algorithms have emerged [16]. The ultimate aim of machine ethics is to effectively investigate how to design intelligent machines to reason morally and ethically. It is concerned with how intelligent machines behave towards humans and other autonomous machines. The main goal of machine ethics is to develop an intelligent machine that makes decisions about potential courses of action under the guidance of an acceptable ethical dimension. It is important to distinguish between an implicit and explicit ethical machine [17]. An implicit ethical machine means constraining the intelligent machine’s actions to avoid unethical outcomes. One practical technique for achieving this is by developing a software system’s internal functionalities and features to implicitly support and promote ethical behavior [14]. On the other hand, explicit ethical machines can explain ethical information by using explicit representations of ethical principles [14,18]. Explicit ethical machines can handle new situations and reasonably make explicit ethical judgments [14,18].

The ML research community has begun to explore the application of modern ML capabilities to machine ethics. Various ML approaches to ethical reasoning have previously been introduced. For example, the work in [19] explores a neural network model that classifies specific ethical judgments and case-based moral reasoning. A case-based reasoning approach to developing systems that can guide reasoning about ethical problems and dilemmas is briefly described in the work in [20]. One of the main questions raised in [20] is how machines can assist or potentially take humans’ place in ethical reasoning.

A different approach to computing ethics that adopts an action-based approach to ethical theory is presented in [21]. The authors developed an efficient decision procedure for an ethical theory that has multiple computing duties [21]. In addition to the ML capabilities, there are other approaches to this problem, for example, using deontic logic (the field of philosophical logic is concerned with the notion of obligation, permission, and related concepts). For example, the authors in [22] described how deontic logic can be used to incorporate a particular set of ethical principles into the decision-making process of autonomous systems. On the other hand, the work in [23] evaluates the viability of applying deontic logic approaches to implement the fundamental principles of Immanuel Kant on categorical imperative and moral obligation. As a general approach of Immanuel Kant on machine ethics, a decision procedure exists for generating categorical imperatives from which rules of action are derived. According to the results of the approach presented in [23], the deontic categories are formulated as forbidden, permissible, or obligatory actions.

### 2.3. Properties of Autonomy

The literature indicates multiple approaches to defining the notion of autonomy and autonomous systems in the context of distributed AI. Autonomy can be defined as the ability of an intelligent agent to act independently without direct external intervention and make decisions with minimal human supervision. The definition of autonomous systems concepts also varies in terms of their autonomy properties. Its external and internal states determine the properties of autonomy. A system can be considered autonomous when it acts non-deterministically. Non-deterministic systems may exhibit different behaviors even for the same environmental inputs of an identical situation or may even fail completely. On the other hand, an autonomous system may also be deterministic if the internal state of the system is taken into account. A deterministic system is a system whose models consistently yield the same result from a given environmental initial state or situation. In this context, pro-activity, interaction, and emergence are the three properties that best describe autonomy and its relevant underlying characteristics [24,25,26]. A summary of the properties of autonomy is shown in Table 1.

**Pro-activity**. Intelligent autonomous systems must safely adapt to unanticipated situations in a dynamic and unpredictable environment to be used across a variety of domains [27]. When the autonomous system activates goals or initiates actions without explicit external events, this property of autonomy is referred to as pro-activity [24,25,26].

**Interaction**. This property refers to the interaction of an intelligent agent with the environment. An autonomous system can dynamically interact and respond to a complex and unpredictable environment. In addition, intelligent autonomous systems can also adapt to changes in the dynamic environment. This property is important in real-time applications [24,25,26].

**Emergence**. Complex multi-agent systems are made up of multiple interacting subsystems. The interaction and pro-activity nature of intelligent agents produce emerging autonomous properties that are not explicitly modeled in advance. Emergence in the context of large-scale multi-agent systems is characterized by an unexpected system behavior caused by nonlinear interactions with the environment over time. This property impacts system reliability and predictability, and it is used as a criterion to evaluate autonomous software systems [24,25,26,28].

### 2.4. Regulation and Levels of Autonomy

**Regulated Autonomy**. As the current advancements in AI research and the impact of modern autonomous systems are becoming pervasive, it is very important to establish policies, regulations, and guidelines to ensure that AI-driven intelligent systems remain trustworthy, ethical, and human-centric. For example, the privacy regulations adopted by the European Union’s general data protection regulation (GDPR) [29,30] and the United States’ Fair Credit Reporting Act (FCRA) [31] give directions on how personal internet data should be processed and grant individuals the right to access their personal information and receive reasonable explanations about decisions made by intelligent automated systems. Adopting a set of regulations such as these enables us to assess the legal and ethical concerns around AI-driven autonomous systems and the way they operate.

**Levels of Autonomy**. According to previous research works, the levels of autonomy are classified into strong regulation, operational autonomy, tactical autonomy, and strategic autonomy. The mappings of levels of autonomy to the properties of the underlying dynamic environment are described in [26]. The properties of the environment include observable, deterministic, episodic, static, and agents. An observable environment has full or partial access to all the required states of the system at all times. A deterministic environment is one in which the next state of the underlying environment is completely determined by the current state and the actions selected by the agents [32]. The agent’s experience is divided into multiple independent episodes in an episodic environment. Each episode in the environment consists of the agent perceiving and then acting. In other words, an episodic setting is where the previous action does not affect the next observation [32]. However, if the subsequent action depends on the previous action, the environment is referred to as sequential. If an environment does not change over the passage of time, it is referred to as static. An environment is called dynamic if it changes while processes are operating on it. A single-agent system means only one agent acting and interacting in a specific environment. However, if multiple interacting intelligent agents interact with one another and their environment, it is referred to as a multi-agent system.

Strong regulation represents systems with no autonomous capabilities. Such regulations are effective in environments with limited complexity. Operational autonomy represents the operational level of decision-making. Intelligent software systems implementing operational autonomy are practically effective in environments that are partially observable, deterministic, episodic, and static [26]. Tactical autonomy extends operational autonomy in the context of tactical decision-making of autonomous systems.

## 3. AI Techniques for Tactical Autonomy Capabilities

Autonomy is an active area of research in both academia and industry sectors. With the proliferation of modern distributed autonomous systems and smart technologies, AI and ML approaches have significantly advanced the state-of-the-art for various research domain problems. AI approaches have a critical role in drastically improving the performance and safety of autonomous systems. Fully autonomous and other complex networked systems are configured and programmed to operate continuously. These sophisticated systems constantly collect complex information from the surrounding environment. Hence, operating and understanding the dynamics and kinematics of fully autonomous systems and processing the enormous flow of information in real time is extremely challenging and beyond human capability. This is when AI-based technologies and their underlying ML capabilities are overwhelmingly helpful. AI and ML systems have proved to be more powerful and efficient than humans in several domains [33,34,35,36]. In addition to this, AI and ML systems often guide human understanding and autonomous decision-making processes in complex situations [37,38].

Advanced AI and autonomous system technologies have already changed our lives and will continue to change in the future. The potential for this unprecedented success of the AI-enabled technological revolution is the rapidly increasing applicability of AI systems across various emerging technologies. For example, over the last decades, AI techniques have created potential real-world impacts in the robotics and autonomous systems community. In addition to the potential benefits of AI, there are also concerns about the longer-term implications of robust AI systems [39,40,41]. Recent advances in powerful AI and ML techniques for tactical autonomy have revolutionized a wide range of areas, including autonomous driving [42,43,44], the aviation and aerospace industries [45], unmanned aerial vehicles (UAV) navigation [46], maritime defense [47,48], etc. Most recent approaches for autonomous systems are based on different techniques of AI. A summary of the state-of-the-art AI techniques for tactical autonomy is presented in Table 2. Some of the main classes of approaches in detail include the following.

**Deep Learning (DL)**. This is an effective and powerful algorithm for AI applications, such as computer vision, natural language processing (NLP), robotics, AI-enabled games, and other applications. Since their inception, deep learning (DL) approaches have proven to be effective at discovering and learning the complex structures of high-dimensional training data [49]. Due to the tremendously promising performance brought by deep neural models in complex environments, DL techniques have recently been used to solve several real-world applications, such as autonomous driving [50,51,52], computer vision [53], image classification [49], video prediction [54], etc. The authors in [55] demonstrated how a deep Q-network (DQN) agent could learn to make a general-purpose tactical decision model for autonomous driving. DL approaches also help predict the behavior and performance of an autonomous vehicle in complex driving settings based on the current and past observations of the surrounding environment [56,57]. Furthermore, an approach that estimates end-to-end lane positions using a deep neural network is presented in [58].

**Reinforcement learning (RL)**. To realize the full impact and potential of AI techniques requires intelligent autonomous systems’ ability to learn and automatically make independent decisions on their own dynamically. A fundamentally different approach to tactical decision-making tasks relevant to autonomous systems is to utilize an AI/ML technique that does not require the input training data to be labeled. One powerful ML paradigm for accomplishing such tasks is applying reinforcement learning (RL) techniques [59]. RL is a framework that provides an efficient solution to experience-driven sequential decision-making problems [59]. It is concerned with how intelligent AI agents should make suitable decisions in a complex and noisy environment to maximize the cumulative reward of a particular executable action. RL is based on a sequence of dynamic interactions between a self-learning AI agent and its complex environment. Through its self-learning capabilities in AI agents, RL is enabling exciting advancements in various domains of science such as autonomous robotics [60], autonomous driving [61,62], NLP [63,64], game playing [65,66], and many other applications. RL techniques can be utilized to create a general tactical decision-making agent for autonomous systems. For example, Bayesian RL techniques based on an ensemble of neural networks are employed for effective tactical decision-making agents for autonomous driving [67]. Moreover, some recent works have also extended deep RL-based techniques for autonomous navigation tasks in mobile robotics [68,69].

**Federated learning (FL)**. In traditional ML and DL applications, training data from different clients are typically aggregated in a central server or a cloud platform to train the model to effectively perform a given task [70]. This is a common data privacy issue, and it is the fundamental limitation of classical ML and DL methods, mainly when the training data contains highly sensitive and classified information (e.g., national secrets and military-related information, hospitals, etc.) that can raise broad security and privacy as well as legal and ethical issues. Maintaining the security and privacy of intelligent systems remains an open challenge. This is the situation when federated learning (FL) technology is helpful. FL is an emerging and promising decentralized ML paradigm that offers a solution by employing distributed computation to address data security and privacy concerns [71]. It enables many resource-limited distributed clients in a network to collaboratively train ML models without communicating their local data with the main aim of protecting the privacy and security of users [72,73,74]. By leveraging interdisciplinary techniques and technologies, robotics and autonomous systems are becoming increasingly ubiquitous. Given the distinctive advantages such as privacy preservation, decentralized learning, parallel training, and onboard processing, FL has the potential to be a secure and efficient AI framework for distributed autonomous systems [75]. In [76], for example, the authors have presented an FL framework that enables collaborative learning of the autonomous controller model across a group of connected and autonomous vehicles. Other authors in [77] have demonstrated that FL models can be utilized to detect and identify different types of UAVs from a larger pool of devices by exploiting radio frequency signals transmitted by individual UAVs.

**Table 2:** Summary of AI techniques for tactical autonomy capabilities.

References	Key Ideas	Category
Ref. [55]	Demonstrated how a DQN agent could learn to make a general-purpose tactical decision model for autonomous driving.	DL for tactical autonomy.
Ref. [59]	An advanced approach to tactical decision-making utilizing RL techniques.	RL for tactical autonomy.
Ref. [60]	Advancements of autonomous robotics using RL-based techniques.	RL for tactical autonomy.
Refs. [61,62]	RL-based techniques for autonomous driving.	RL for tactical autonomy.
Refs. [63,64]	Solving different problems of NLP using RL.	RL for tactical autonomy.
Ref. [75]	Secure and efficient AI framework for distributed autonomous systems.	FL for tactical autonomy.
Ref. [76]	FL framework that enables collaborative learning of the autonomous controller model across a group of connected and autonomous vehicles.	FL for tactical autonomy.
Ref. [77]	Demonstrates that FL models can be utilized to detect and classify different types of UAVs from a pool of devices by exploiting radio frequency signals transmitted by individual UAVs.	FL for tactical autonomy.

## 4. Trusted AI and Mission Autonomy

State-of-the-art AI and ML techniques are being increasingly employed in a wide array of time-critical and safety-critical systems that require improved operational assurance, such as military, defense, aerospace, autonomous driving [78], medicine [79], science [80] etc. To enhance and ensure their end-to-end effectiveness and resilient operations, these modern autonomous systems with AI capabilities must be continuously validated, verified, and monitored. Furthermore, a continuous system performance evaluation that recognizes unforeseen risks, anomalies, and potential adversarial threats is required for autonomous systems to maintain a robust operation. Moreover, there are also AI-enabled military concerns regarding autonomous weapons beyond human control [81].

**Figure 2:**
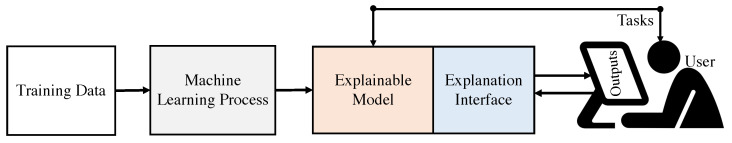
Explainable AI. As presented in Section 8, developing advanced ML techniques to produce explainable models is one direction of our future work. In addition to this, integrating state-of-the-art explanation interfaces that produce efficient explanations of the underlying models is a challenge we plan to explore in our future work.

**Explainable AI**. Recent advances in ML techniques have led to growing interest in the explainability of AI systems to help humans gain a deeper insight into the decision-making process of ML algorithms. The widespread deployment of advanced AI systems across various complex applications over the last few years has been coupled with a rise in ethical, legal, and societal demands for these systems in providing human-understandable model explanations and interpretations for their outputs. As a result of these demands, several recent works on a regulation requiring explanations and interpretations of the decisions made by AI-based automated systems have been introduced [82,83,84]. This has also led to a growing research community with a strong focus on explainable ML techniques. As shown in Figure 2, providing users with understandable explanations and interpretations allows them to gain deeper insight into the system’s automated decision-making perspective, which is the key element in establishing trust in the underlying AI and ML systems [85,86,87]. Hence, building explainability and interpretability into AI models and techniques of critical systems also create impacts on safety [88], ethics [89,90,91], law [92,93,94], and transferability [95]. However, the inner workings of AI and ML systems are difficult to understand by human beings and are considered black-box methods where only inputs and outputs are visible to users [96]. This lack of algorithmic transparency in AI and ML systems, lack of understanding of real-world user needs, and our inability to adequately explain how and why these systems reach particular AI-driven automated decisions make it fundamentally difficult to understand, even by experts in the field [96,97]. For humans to fully trust and build confidence in AI-powered systems, the explanations of the underlying system must be consistent with human expectations and perceptions. Recently, an increasing variety of open-source explanation tools and platforms that produce different explanations for the exploration and interpretation of the underlying black-box ML models are being accessible to users [98,99,100,101]. However, despite recent efforts, most of the current state-of-the-art techniques of explanation and interpretation need to be more trustworthy.

**Figure 3:**
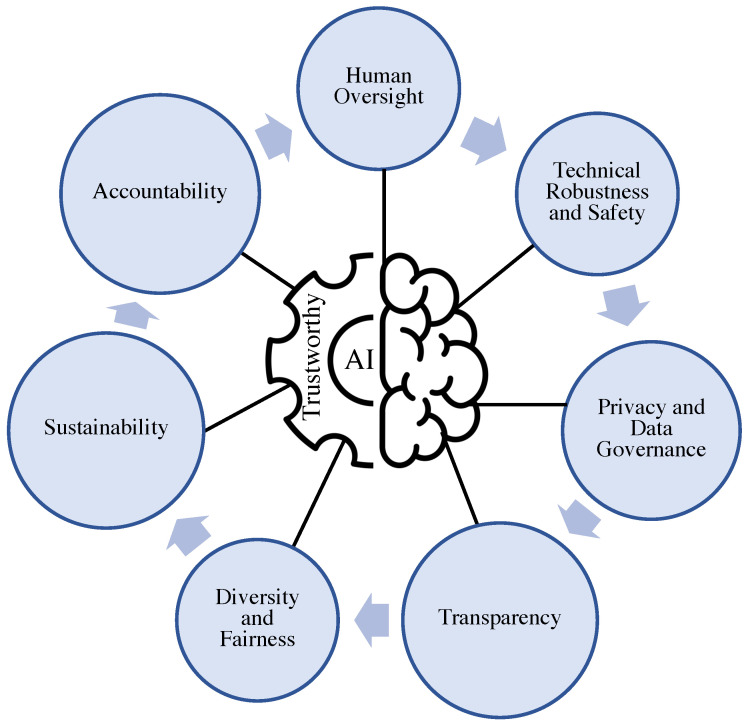
Requirements and elements of a trustworthy AI [108].

**Trustworthy AI**. Advanced AI and ML models enable accelerating data-driven automated decision-making processes in complex systems. However, as explained earlier, despite the recent widespread adoption of AI and ML systems in science and technology, their system models remain largely black-box methods. Having a clear and full understanding of how these complex systems fully operate is useful in establishing trust and transparency. In addition, understanding the inner workings of AI and ML systems gives users a better insight into the underlying model, which can then be utilized to transform a model from untrustworthy to trustworthy. Determining the trustworthiness of AI and ML models is a fundamental problem when the model is utilized for automated decision-making systems. As explained in Section 6, the collaboration between humans and intelligent machines has enabled the rapid advance and wide use of modern autonomous systems. The effective use of such complex systems in the military and national intelligence agencies and other critical domains depends on the trust established between humans and machines. Therefore, given the rapidly expanding applicability of AI-driven technologies in numerous autonomous systems, it is more important than ever to make these systems reliable and trustworthy [102]. Building a safe and trustworthy AI ecosystem is crucial for ensuring human safety and adopting advanced AI-enabled technologies across various applications [103]. Trustworthy AI is a technical term that describes the safety, legality, robustness, and ethical principles of AI, including fundamental concerns with security [104], privacy [105], transparency and fairness of AI-powered systems [106,107]. The requirements and elements that make AI systems trustworthy are shown in Figure 3. The fundamental concept of trustworthy AI is based on the notion that AI reaches its full potential when trust is established. Trustworthiness gives AI-enabled systems explainability techniques that make it easier for humans to understand and trust the characteristics and reasons behind the results and outputs produced by the ubiquitous AI algorithms.

**Mission autonomy**. It is a technical term mostly used in the defense and aerospace technology industries and other next-generation autonomous and intelligent systems. Mission autonomy is the ability of an autonomous system to independently execute a variety of fundamentally complex tasks, e.g., deep space exploration missions, based on the knowledge and understanding of the underlying system using modern data-driven AI/ML techniques [109]. For the development and implementation of advanced mission autonomy systems to be tactically useful, it is important to address the potential security and risk issues associated with autonomy and AI systems described above.

## 5. Collaboration between Platforms

The proliferation of advanced algorithmic decision-making systems has enabled the collaboration of different platforms. However, enabling and determining direct collaboration between humans, intelligent machines, and autonomous agents is challenging. Some of the main technical challenges that need to be addressed are interoperability, composability, and adaptability.

**Interoperability**. In the context of autonomy, interoperability enables different kinds of large-scale autonomous systems to communicate independently through the underlying platforms. Interoperability issues occur at different levels when designing interacting autonomous agent systems with a strong notion of autonomy. As described in detail in [110], interoperability layers can be classified as connection, communication, ontological, and service layers.

**Composability**. In the world of software systems development, composability is necessary for creating a robust, flexible, and interoperable system where different interacting autonomous components communicate seamlessly [111]. It enables combining independent functionalities of a component-based system to accomplish a given global task that could not have been accomplished independently. Composability gives system designs the ability to increase agility by reusing existing system components and adapting to new changes [111]. A composable architecture allows the assembly of several system components. An approach such as this has important benefits, including reusability, flexibility, and improved modularity. Autonomy, modularity, and discoverability are the main elements of composable components. Each component in a composable system is expected to autonomously and independently perform a given task without the assistance of other components. Modularity, on the other hand, refers to the property of a system when each component in a composable system is designed to solve a specific task independently. This makes it possible for system designers to assemble modular components into one system. In addition to this, the frameworks of the composable system must be discoverable by other users in order for individual components to be reused.

**Adaptability**. An interactive autonomous system needs to be aware of its internal state and the complex environment where it robustly operates. Advanced autonomous systems have the ability to autonomously and interactively monitor and adapt to any unexpected changes in a complex environment. The degree to which a complex system efficiently deals with a dynamic functionality change in operating environments is referred to as adaptability [112,113]. An adaptable, robust, and resilient system tolerates sudden changes and dynamic situations in an environment without relying on external intervention [112].

## 6. Human–Machine Teaming

The concept of human–machine teaming and its capabilities are at the core of many current advances in AI research. Human–machine teaming is a paradigm in which humans and intelligent machines with different capabilities integrate and closely work together to accomplish a common goal that requires collective action [114,115]. It is concerned with the deep understanding and evaluation of intelligent machines intended for human use [116]. Given the recent exponential growth and the predictive capabilities of AI technologies, creating a successful collaboration in the operating environment between intelligent systems and humans to solve complex problems is crucial. However, one of the main challenges to the widespread adoption of AI systems is the ability to seamlessly integrate humans and distributed intelligent systems to achieve a common goal.

The effective exploitation of human–machine teaming enables humans to gain a deeper insight into the automated decision-making of intelligent machines. However, as explained in Section 4, this highly depends on the trust between the AI-enabled automated decision-making systems and humans. This is because when humans place more trust in AI-powered decisions, it raises questions about trust issues. The effectiveness of human–machine teaming mainly depends on the transparency of the machine and the level of user confidence that AI systems will behave as expected, securely, safely, and understandably [117]. Broad collaboration across multiple disciplines, autonomous systems powered by modern AI techniques, and domain experts is very compelling for establishing the explainability of AI/ML models, creating a trustworthy AI ecosystem, and unlocking the potential of AI to solve more significant problems.

AI has the potential to improve human capabilities, automate organizational decision-making, and fundamentally transform the way businesses operate [118,119]. The explainability of AI/ML systems is a potential approach for human–machine teaming since automation with the capability to explain and interpret results enables humans to understand the underlying behavior of intelligent machines better. One of the main benefits of using an autonomous system is the ability to process more data in real-time much more quickly than a human can. To ensure security, safety, and effective mission-critical operations, autonomous systems across various domains, such as defense, healthcare [120], aerospace, manufacturing, autonomous driving, etc., are evaluated to operate collaboratively with humans. Therefore, exploring cutting-edge techniques for better human–machine teaming has the capability to enhance productivity, usability, reliability, operational performance, communication interface, cost of designing and operating platforms, share knowledge between humans and the intelligent machines, and ensure safety and the ability for existing systems to adapt to new environments and new tasks [121,122]. A human–machine teaming framework that guides AI development teams to create broadly adopted ethical AI systems that are usable, secure, and trustworthy is presented in [123]. In addition to this, major players, such as IBM [124], DeepMind [125], Google [126], and other academic institutions recently initiated a research effort to enhance human–machine collaboration [127,128,129].

### 6.1. Ad Hoc Human–Machine Teaming

Significant advances in autonomous systems are increasingly enhancing the quality of our daily lives. Given these technological advances over the past few years, different forms of human–machine teaming have emerged. Ad hoc teaming is the process through which humans and intelligent machines with varying knowledge and capabilities collectively collaborate to achieve a common goal [130]. Ad hoc human–machine teaming is a challenging scenario where an intelligent agent collaborates with unknown heterogeneous teammates without prior knowledge of coordination. An effective ad hoc team player is an agent skilled at evaluating other agents’ capabilities in comparison to its own capabilities. Effectively and robustly collaborating with heterogeneous teams on the fly without any pre-condition is important in the military, industrial, and other autonomous settings. Collaboration without any prior coordination is a known challenge in human–machine research [131]. As an approach to address this problem, an online planning algorithm for ad hoc team settings designed for situations where agents collaborate without any pre-coordination is presented in [132].

### 6.2. Challenges Associated with Current Human–Machine Teaming Approaches

The following are some of the main challenges that limit our ability to effectively integrate humans and intelligent machines in a dynamic operating environment.

**Heterogeneity**. In human–machine teaming, it is difficult for the intelligent machine to predict and adapt to human actions in the face of dynamic operating environments due to the significant heterogeneity in human decision-making tasks. Therefore, it is important to develop state-of-the-art models and techniques that can be used to address the issue of heterogeneity in a human–machine teaming setting.

**Communication**. The success of human–machine teaming depends on effective communication between humans and intelligent machines. Humans have limited communication capabilities and can only process a finite amount of information. Therefore, by simply exchanging essential information, humans and machines can effectively communicate information that supports human–machine teaming. However, this creates trust problems between humans and machines. A key component of effective team communication is the trust established between intelligent systems and humans [133]. In human–machine teaming, trust is defined as the user’s confidence in the reliability of the intelligent system’s conclusions and its ability to accomplish a defined goal [134,135]. The concept of transparency is a key aspect of information exchange since humans and intelligent machines require shared knowledge and a common understanding of intent, the reasoning and decision-making process, performance, and future plans [136,137].

Communication may help establish trust when humans and machines work together as teams. Additionally, it can be used to establish guidelines for the efficient design of the information that promotes overall performance and trust of human–machine teaming [138]. However, machines must first be able to roughly mimic how humans process information for the machines to exchange information in a way that humans can understand it. A human–machine teaming relationship has three most important components: the human, the intelligent machine, and the interactions between humans and intelligent machines (or alternatives). Hence, as discussed above, establishing trust through developing an explainable and trustworthy AI is crucial to the success of human–machine collaborations. However, AI systems’ growing complexities and vulnerabilities and their ability to learn and adapt to dynamically changing operating environments also raise new challenges in establishing trust in human–machine teams.

**Coordination**. To fully maximize the potential of a heterogeneous team, humans and intelligent machines should collaborate in an efficient and coordinated manner. As explained above, communication in the context of human–machine teaming refers to exchanging information between humans and intelligent machines or alternatively. Coordination, on the other hand, refers to the organization and management of team members and their associated behavior to achieve a specific common goal [139,140]. According to [141], effective human–machine coordination involves three basic requirements. These requirements are *common ground*, *interpredictability*, and *directability*. In order to communicate accurately and effectively as a team, participants must first identify the appropriate common ground, i.e., knowledge, mutual beliefs and assumptions, shared goals, etc. Common ground refers to information that is mutually believed by all participants involved in a conversation [141]. Whereas the ability of the coordinating team members to reasonably predict each other’s actions and behaviors is referred to as interpredictability [141]. Directability, on the other hand, refers to the ability of the team members to redirect, help, or influence each other’s behaviors when circumstances and priorities suddenly change [142]. Hence, developing an advanced model that supports implicit coordination based on these three requirements is important. Implicit coordination is defined as the process of synchronizing the actions and behaviors of team members based on assumptions and intent without using behavioral communication [143,144]. This means communication is not necessarily mandatory for implicit coordination. Implicit coordination helps increase team effectiveness because it makes it possible for team members to work together by avoiding distraction and communicating effectively even when direct communication is not available [145]. This, in turn, significantly reduces communication overhead [146].

**Adaptability**. The ability to effectively change a course of action in reaction to unexpected changing, complex conditions by adjusting strategies and behaviors is called adaptability [113]. Adaptability can be divided into two categories: human-assisted adaptability, and machine-assisted adaptability [147]. Intelligent machines should be able to recognize the knowledge and behaviors of human teammates. In addition, machines should also be able to predict and respond to new knowledge and behaviors of humans. However, this requires the development of modern adaptive (i.e., machine-controlled adaptation) and adaptable (i.e., human-controlled adaptation) systems.

## 7. Cybersecurity for Tactical Autonomy

Autonomous systems have attracted a great deal of attention in recent years from the academia and industry sectors. However, the widespread and effective adoption of autonomous systems across a wide variety of domains also poses a significant increase in security attacks that needs to be addressed. Because cyber attackers aim to target large-scale autonomous systems such as modern autonomous vehicles (AV), crewed spacecraft, space traffic management systems, ships, mobile robots, operations of complex nuclear plants, aircraft, critical infrastructures of smart cities, etc. to compromise the safety of the system and cause disruptive damage to their operations. Therefore, it is crucial to design AI-based approaches that proactively respond to potentially disruptive attacks that attempt to compromise and gain access to autonomous systems and their command components, for example, by targeting the underlying autonomous decision-making capability of the systems. Automatically detecting and responding to overwhelming volumes of security threats, handling vast amounts of data, and discovering new patterns of unknown attacks are some of the benefits of AI systems for cybersecurity [148].

**Challenges of AI in Cybersecurity**. AI can introduce unforeseen legal, ethical, and societal risks and challenges that, if not effectively addressed, may significantly reduce its potential. As discussed above, AI and its advanced ML techniques have evolved into an enabling technology for a wide range of innovative and dynamic domains. AI has both tactical and strategic potential benefits. However, it is also perceived to have some critical constraints and limitations in the context of trust and ethical considerations associated with using AI systems. For example, the authors in [149] addressed that AI itself may pose a threat to cybersecurity and legal and ethical concerns. They argue that the lack of interpretability and explainability in AI systems can be leveraged to hide security attacks [149]. Another work in [150] has also demonstrated that AI has both positive and negative consequences regarding cybersecurity threats. Moreover, in light of the rise of AI-driven cyberbullying, the authors have also argued that cybersecurity experts should be allowed to continue doing their job and conduct network testing when human intelligence is necessary.

### 7.1. Intrusion Detection

Intrusion detection systems are designed to detect intrusions or security attacks in a network that unavoidably occur despite precautions [151]. There are various approaches to intrusion detection systems. Some methods employ a signature-based technique in which events are detected and compared against a predefined database of signatures of known security attacks and intrusions [152,153]. Other systems employ anomaly detection techniques where the systems find potentially harmful patterns in data that do not comply with expected notions of normal behaviors [154,155,156]. In modern autonomous technologies, it is equally important to monitor and identify anomalies, detect illicit and malicious activities, and take remedial actions to ensure sustained operations of real-time autonomous decision-making systems, especially in tactical environments. A prototypical distributed intrusion detection architecture implemented that uses autonomous agents tailored for tactical environments is proposed in [157]. An AI-based approach for identifying and detecting intrusions in UAVs is proposed in [158].

### 7.2. Anti-Autonomy

Anti-autonomy technologies are increasingly gaining popularity and various approaches have previously been proposed to address this problem. When an autonomous system’s underlying confidentiality and functionality are compromised, it makes itself more vulnerable to future security attacks and poses a potential threat to other autonomous systems. Therefore, it is critically important to proactively detect and identify potential cyberattacks that aim to target autonomous systems under continually changing conditions. In [159], the authors investigate the security and privacy challenges that need to be addressed to increase the resilience of cyber-physical systems. An intrusion detection system for self-driving cars is presented in [160]. The work in [160] addresses that an autonomous vehicle, if compromised, could also pose a risk to passengers and pedestrians on the roadway. In addition, their paper discusses how interconnected self-driving car vulnerabilities go beyond just endangering drivers, passengers, and pedestrians on the roadway. The authors argue that the coordination of interconnected autonomous vehicles could potentially be used to launch a wide-scale attack that affects a large-scale vehicular ad hoc network (VANET) [160].

UAV systems have immense potential to revolutionize research and innovation across a broad range of next-generation technological applications. Such systems could potentially be vulnerable to sophisticated attacks aiming to compromise their complex operations and autonomous decision-making capabilities. These attacks could be employed for both offensive and defensive cyber operations. Therefore, it is necessary to develop flexible and proactive strategies that effectively provide a potential defense mechanism against attacks that aim to exploit vulnerabilities in safety-critical autonomous systems in real time under minimal human control.

## 8. Some Challenges Related to Tactical Autonomy

Tactical autonomy offers a good solution for many defense and military applications with limited human involvement. ML and AI systems have created unprecedented opportunities for achieving autonomy for civilian and military applications. However, to develop long-term, trustworthy, robust, and safe autonomous systems, fundamental challenges need to be addressed. A practical understanding of the complex techniques and technologies used in intelligent systems is a critical part of many AI and ML systems that are core components of tactical autonomy.

Although there are many open research problems to tackle, some of the most long-standing and significant challenges that need to be addressed to realize the full penitential of tactical autonomy for defense and other applications include the following.

- *Trustworthy AI for tactical autonomy*. Developing trusted, robust, and resilient AI and ML frameworks for critical defense missions requires an understanding of the theoretical and practical techniques and methodologies related to trusted AI and mission autonomy, the collaboration between platforms, and human–machine teaming enabled by addressing the critical technical challenges discussed in Section 4, Section 5 and Section 6, respectively. To enhance confidence in AI systems, we need to conduct more research to address these issues and make AI systems trustworthy.
- *Verification of AI-based models*. Making sure that AI-based solutions are working as expected is critically important. However, designing state-of-the-art methods for verifying AI-based systems is challenging and takes a lot of work.
- *Collaboration between platforms*. Improving the real-time collaboration between humans and fully autonomous systems (e.g., pilots and autonomous co-pilots) is challenging. Hence, developing an effective and efficient collaborative autonomous solution is a critical challenge that needs to be overcome.
- *Joint human–machine teaming*. It is very important to deeply understanding how machines learn from humans, how humans learn from machines, and how machines and humans work together. How can we design advanced autonomous systems that collaboratively work with humans in military and defense settings?
- *Improving safety*. How do we design and deploy an end-to-end approach that integrates the safety concerns of modern safety-critical autonomous systems?

## 9. Conclusions

The military and defense industry hopes to utilize the capabilities of AI and ML to advance and improve its performance in tactical environments. In this paper, we presented a comprehensive and technical overview of the concepts, techniques, and technologies underlying tactical autonomy. Additionally, our paper highlights some of the critical and operational challenges that arise when attempting to practically build fully autonomous systems for advanced real-world military and defense applications. We, therefore, hope this paper encourages AI and ML researchers to explore further developing architectures and methodologies in the domain of tactical autonomy.

It is significantly challenging to design advanced AI and ML models with practical implications for real-world military and defense applications. Investigating this further with a focus on cutting-edge AI and ML approaches that haven’t been adequately addressed in previous research works is an interesting direction for future work. Further, demonstrating a range of practical applications and state-of-the-art approaches for addressing and gaining insight into some of the long-standing challenges of interest discussed in this paper is another topic for future research directions in the practical applications of tactical autonomy.

## Figures and Tables

**Table 1:** Summary of properties of autonomy.

Properties	Description
**Pro-activity**	A property of autonomy where the autonomous system activates goals or initiates actions without explicit external events [24,25,26].
**Interaction**	An important property in real-time applications that refers to the interaction of an intelligent agent with the environment [24,25,26].
**Emergence**	A property of autonomy produced by the interaction and pro-activity nature of intelligent agents. It is used as a criterion to evaluate autonomous software systems [24,25,26,28].

## Data Availability

Not applicable.

## Abbreviations

The following abbreviations are used in this manuscript:

**Acronym**	**Definition**
AFRL	Air Force Research Laboratory
AI	artificial intelligence
AV	autonomous vehicle
CoE-AIML	Center of Excellence in AI and Machine Learning
DAF	Department of Air Force
DL	deep learning
DoD	Department of Defense
DQN	deep Q-network
DRL	deep reinforcement learning
FCRA	Fair Credit Reporting Act
FL	federated learning
GDPR	general data protection regulation
ML	machine learning
NLP	natural language processing
POMDP	partially observable Markov decision process
RL	reinforcement learning
UAV	unmanned aerial vehicle
VANET	vehicular ad hoc network
